# The Camberwell Infirmary Case

**Published:** 1914-01-31

**Authors:** 


### THE CAMBERWELL INFIRMARY CASE.

### Dr. W. J C. Keats Awarded Damages for Libel.

In the King's Bench Division of the High Court, before Mr. Justice Darling and a special jury last week, Dr. W. J. C. Keats, medical superintendent of Camberwell Infirmary, was awarded £100 damages in an action for libel which he brought against the proprietors of the *London Budget*, and their printers. The defence was that the alleged libel was a fair report of a public meeting, and fair comment on a matter of public interest.

Mr. McCall, K.C., on behalf of the plaintiff, said that in the *London Budget* of March 23, 1913, appeared an article purporting to be a report of a Guardians' meeting containing the following headlines :

"Countess aids flogged boys. Allegations on punishment of convalescent boys in infirmary

"With five-tailed lash.

"Strong complaint before Camberwell Board of Guardians.

"On one occasion a mother waiting in an ante-room was horrified at hearing a lad screaming agonisedly."

He explained that the countess referred to was the Countess de Lormet, a Guardian, and that the woman's name was Bugler.

If there was any screaming, it might have been caused by the removal of a bandage. The protection accorded to newspapers if their report was fair, accurate, and published without malice did not apply to the above headlines. In addition, an interview with the countess was also published in which allegations of the flogging of boys by the plaintiff were made. Four instances were alleged. Before calling Dr. Keats, Mr. McCall's statement that if the defendants would now admit there was no justification in the above headlines and indemnify the plaintiff in costs he would be satisfied, was not taken advantage of by the defendants.

### Dr. Keats' Evidence.

The plaintiff, Dr. Keats, was then called. He stated that convalescing boys were often a little difficult to control, and that it was sometimes necessary to resort to corporal punishment. The strings produced were what he used, and the same with which he used to chastise his own children. In every case he had obtained the approval of the parents. One of the boys he struck three times. The boy did not cry out. Another boy, after warning, had short-circuited the electric light, by which three wards were thrown into darkness, and the electrician had to be called to put matters right. This was a favourite trick of the boys, by which the patients might receive a serious shock. Another boy, who had never been under discipline at home, struck one of the nurses, and ran away, and generally set a bad example. He was brought back, followed by his mother, who said that he was beyond her control, and, on hearing that the witness proposed to whip him before the other boys as an example, said that she was glad. The witness had intended to give him nine strokes, but did not give him so many, and on visiting the boy in the evening said that he hoped what he had done would keep him out of prison. The boy sat up and thanked him. Eventually the boy was sent to an adult ward as incorrigible. He considered what he had done essential in the interests of the boys and for the sake of discipline. The Guardians had approved his report.

Miss Bailey, a sister, Nurse Neal, and Nurse Sutcliffe gave evidence of the unruliness of the boys.

Mr. Randolph, K.C., for the defendants, said that they took a strong view, as they were entitled to, against corporal punishment in an infirmary, and had published a disclaimer which the plaintiff had declined to accept, and that there was not, and never had been, any allegation of cruelty against the plaintiff. The Guardians had not delegated the power to use corporal punishment to Dr. Keats.

After some further evidence had been called, and Mr. Justice Darling had ruled that the matter was one of public interest, the learned Judge summed up.

### Mr. Justice Darling's Summary.

In the course of his address to the jury, Mr. Justice Darling said that to his mind the strongest passage in the alleged libel was the one which stated that "on one occasion a mother waiting in an ante-room was horrified at hearing a lad screaming agonisedly. When the mother asked to see her boy, she was refused permission to enter the ward." They would probably find that that was a libel. It was not disputed that the plaintiff had inflicted punishment with an instrument of five strands, which the newspaper called flogging with a five-tailed lash. In the passage he had quoted it now appeared that the boy who was punished was an orphan, and that the boy whom the mother was not allowed to see was another boy. But would not any reader suppose that it was the mother's boy who was punished? It was obvious that the parents of all the patients could not be admitted to the wards at any time, and that the practices of mischievous boys must be stopped. He was not at all surprised that the Countess de Lormet had taken up the question, which was quite a legitimate thing to do. But it must not be thought that the propriety or otherwise of corporal punishment in the Camberwell Infirmary was being decided. That was a matter for the authorities and the Local Government Board.

The jury returned a verdict for the plaintiff, with £100 damages.

